# Supplementary material for: HDAC1-Mediated lncRNA Stimulatory Factor of Follicular Development to Inhibit the Apoptosis of Granulosa Cells and Regulate Sexual Maturity through miR-202-3p-COX1 Axis
Source: Cells. 2023 Nov 29;12(23):2734. doi: 10.3390/cells12232734 (PMC10706290; doi:10.3390/cells12232734)
Supplement: Supplementary file 1 [file cells-12-02734-s001.zip › Supplementary Tables.pdf]

Table S1 Primers used for vector construction

Primer name	Primer sequences (5’ to 3’)
pcDNA3.1- lnc SFFD	F: CCAAGCTTACTACATGCTTGAGGAGGGTG
	R: GGGGTACCCGCGGTCATACGATTAACCCA
lnc SFFD-WT	F: CGAGCTCACTACATGCTTGAGGAGGGTG
	R: GCGTCGACCGCGGTCATACGATTAACCCA
lnc SFFD-MUT	F: AATGAGAGAGGAAAAGGTCGTCCCTTTCCAGGTATGGC
	R: GCCATACCTGGAAAGGGACGACCTTTTCCTCTCTCATT
COX1-WT	F: CGAGCTCCAAGCACCAGAATCGGACCC
	R: GCGTCGACTCTCAAGGCGGCGAAGAGTA
COX1-MUT	F: TTTCCAACCCATAAGCTACACCTTGACCTAACGTTTTT
	R: AAAAACGTTAGGTCAAGGTGTAGCTTATGGGTTGGAAA
pcDNA3.1-EGFP	F: GGCTAGCCATGGTGAGCAAGGGCGAGGAG
	R: CAAGCTTGTTACTTGTACAGCTCGTCCA
pcDNA3.1-EGFP-MUT	F: GGCTAGCCGTGAGCAAGGGCGAGGAGCTG
	R: CAAGCTTGTTACTTGTACAGCTCGTCCA
pcDNA3.1-ORF+	F: GGCTAGCCACTACATGCTTGAGGAGGGTG
EGFP-MUT	R: CAAGCTTGCGCGGTCATACGATTAACCCA

Sequences underlined represent the enzyme cutting sites

Table S2 Oligonucleotide sequences in this study

Fragment name	Sequences (5’ to 3’)
MiR-202-3p mimic	AGCUACAUUGUCUGCUGGGUUU
MiR-202-3p inhibitor	AAACCCAGCAGACAAUGUAGCU
HDAC1-siRNA	CCGGTCATGTCCAAAGTAA
COX1-siRNA	GATTATCCTGACGCATACA
Si-lnc SFFD	GUAGCCCAUUUCUUUCCAA
