# Supplementary material for: HDAC1-Mediated lncRNA Stimulatory Factor of Follicular Development to Inhibit the Apoptosis of Granulosa Cells and Regulate Sexual Maturity through miR-202-3p-COX1 Axis
Source: Cells. 2023 Nov 29;12(23):2734. doi: 10.3390/cells12232734 (PMC10706290; doi:10.3390/cells12232734)
Supplement: Supplementary file 1 [file cells-12-02734-s001.zip › The uncropped western blot images.pdf]

**Raw images of western blot used in the figures:**

**1.The original blots shown in Figure 1H.**

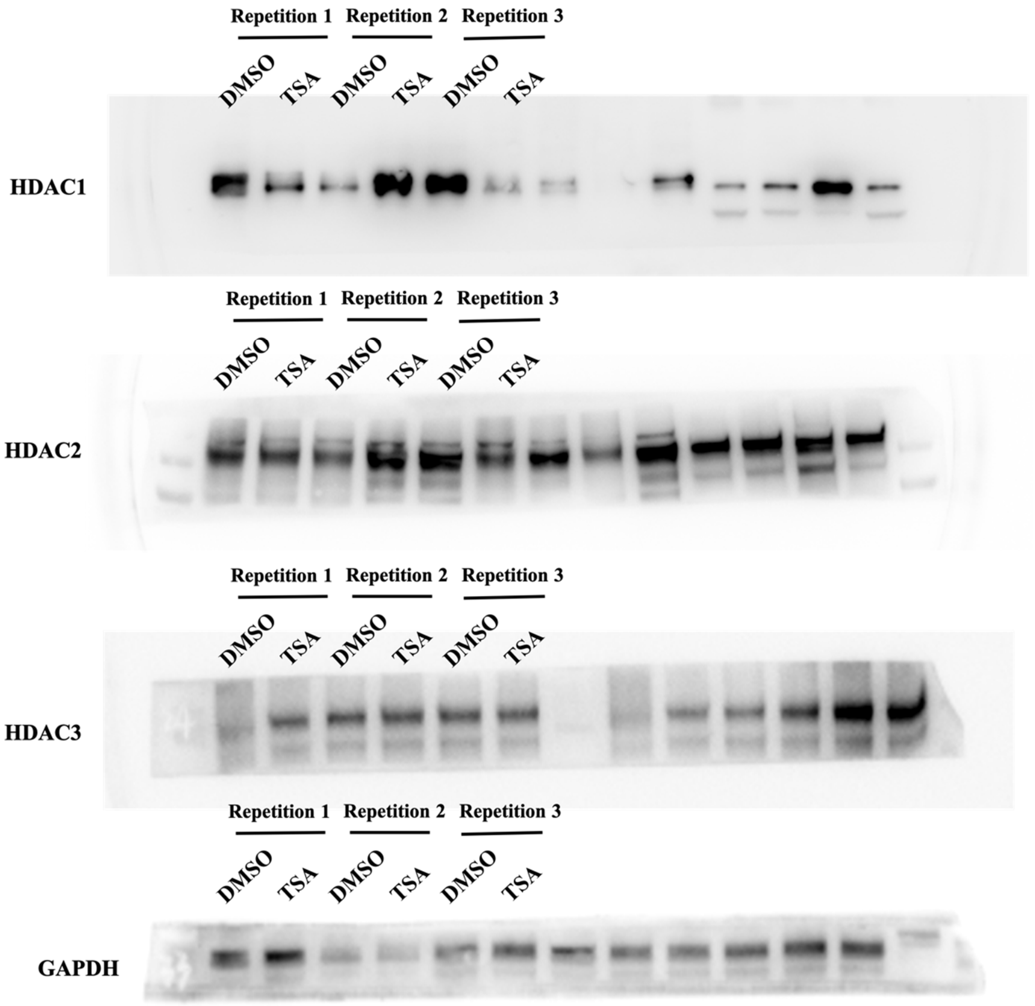

**2.The original blots shown in Figure 2F and 2M.**

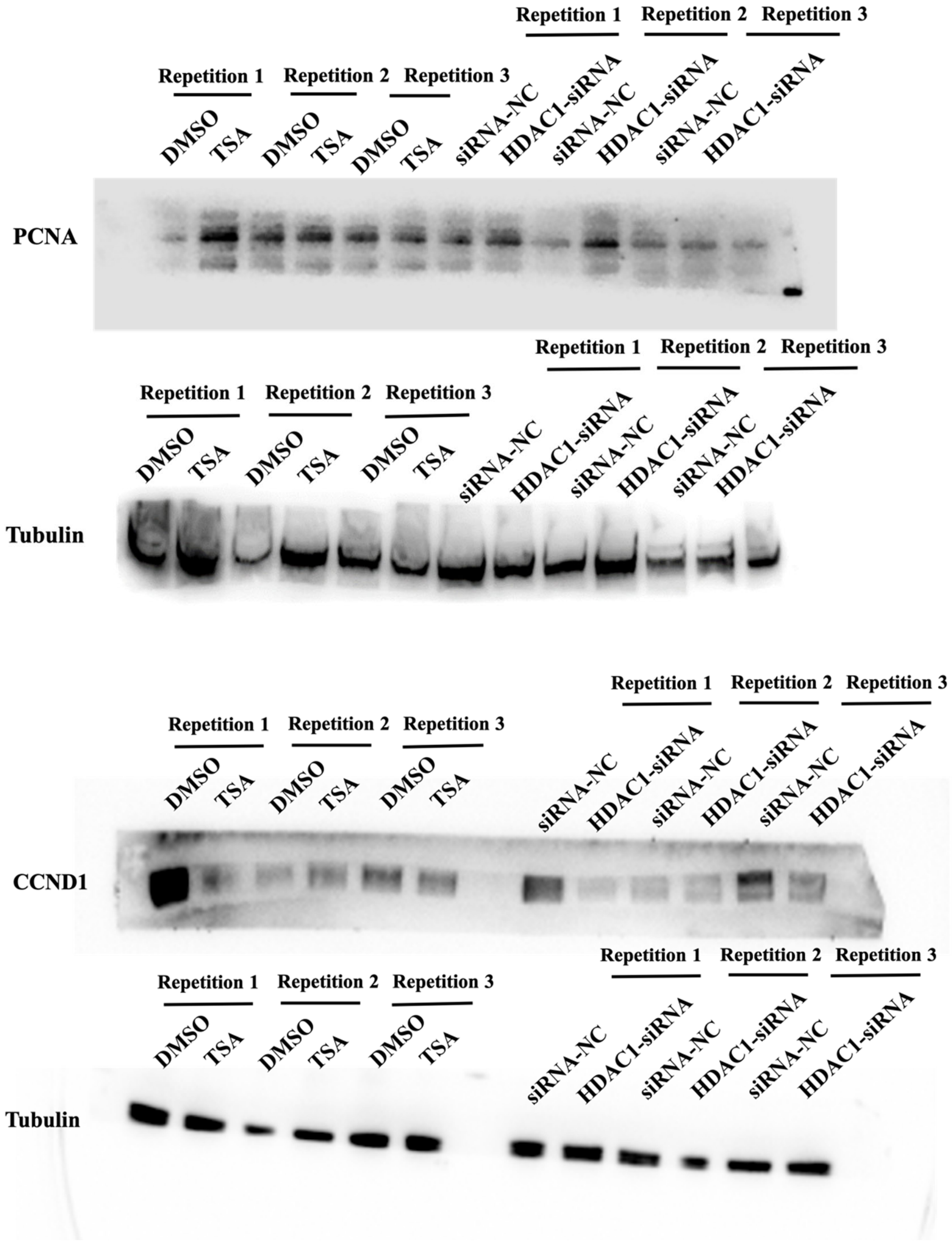

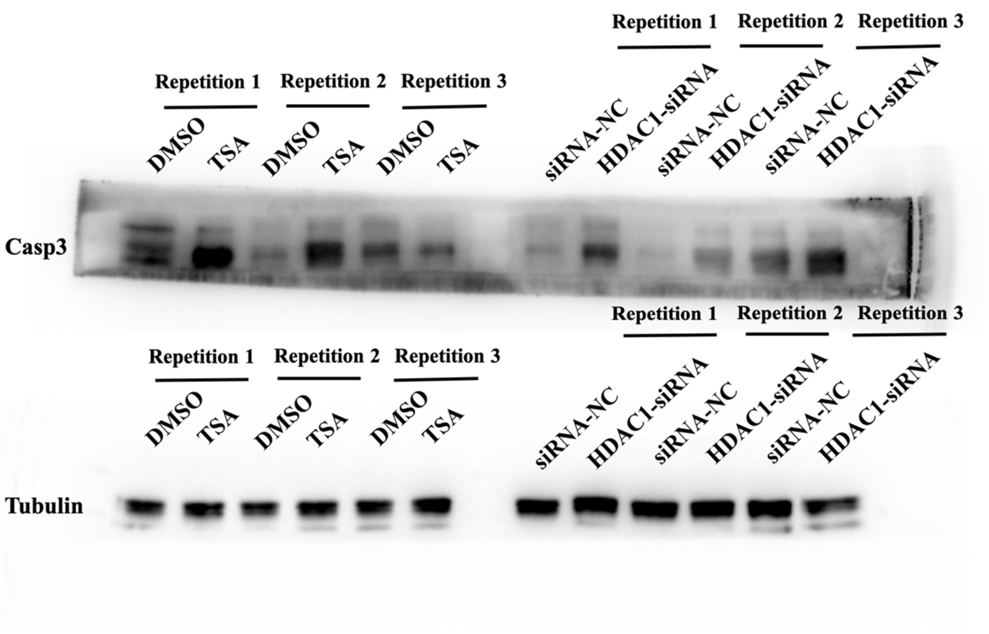


**3.The original blots shown in Figure 2J.**

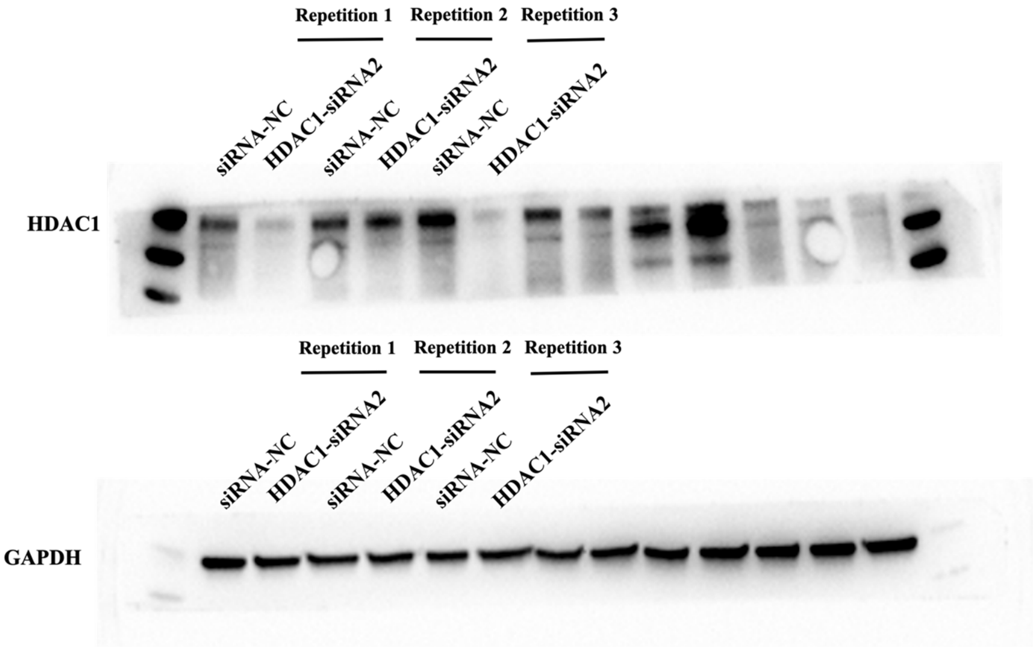

## 4.The original blots shown in Figure 4D and H.

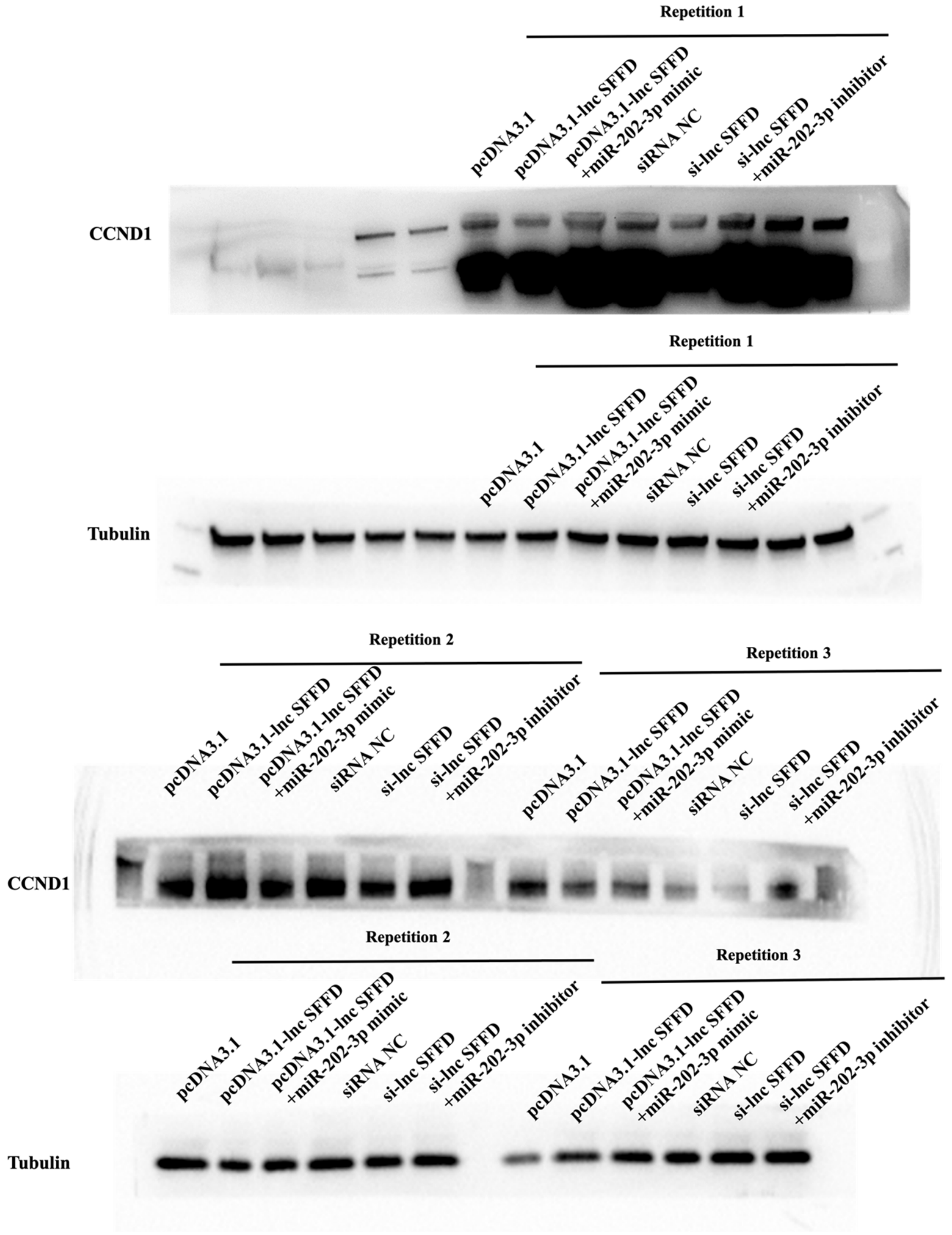

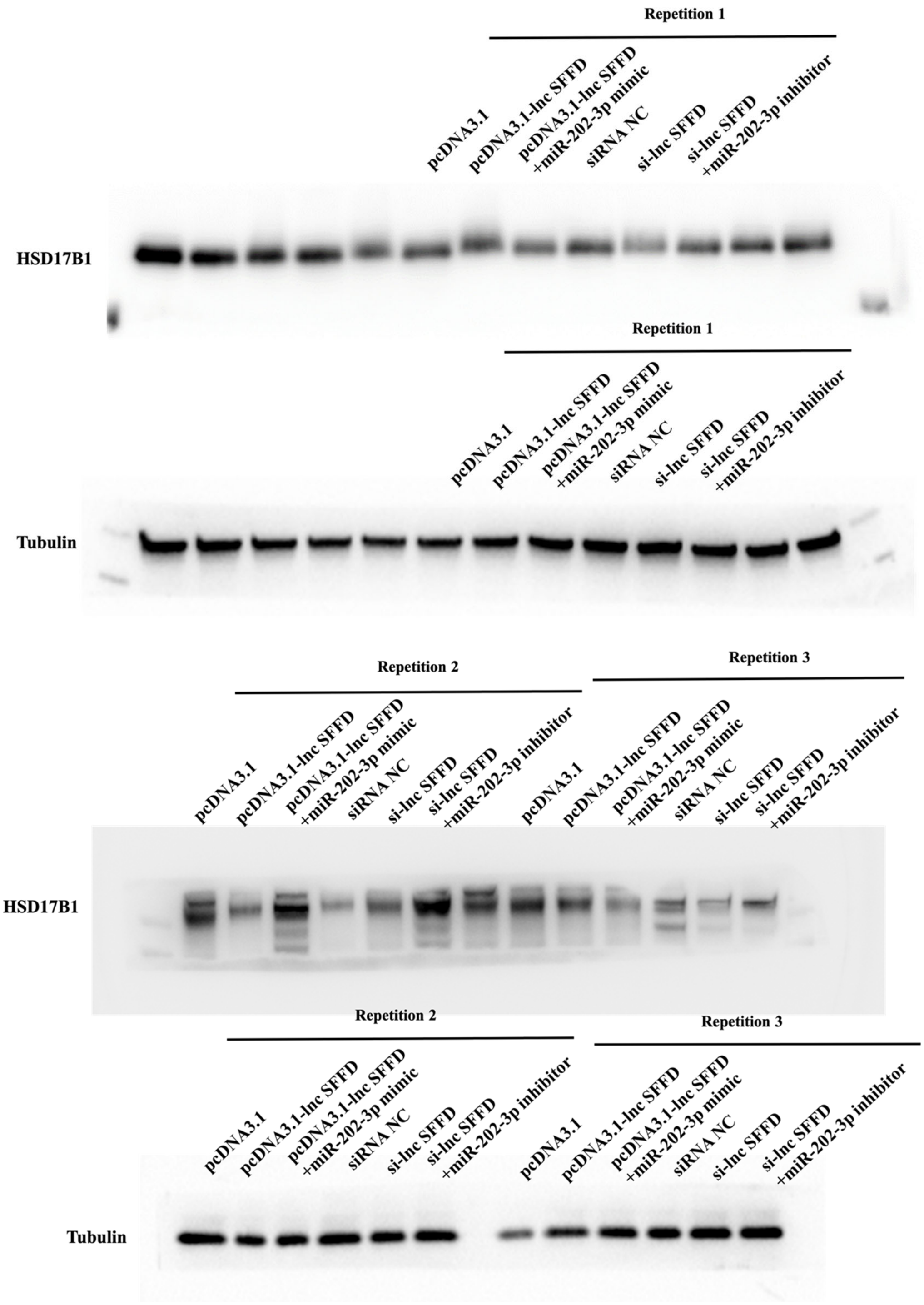
Repetition 1
pcDNA3.1
pcDNA3.1-lnc SFFD
pcDNA3.1-lnc SFFD +miR-202-3p mimic
siRNA NC
si-lnc SFFD
si-lnc SFFD +miR-202-3p inhibitor
HSD17B1
Repetition 1
pcDNA3.1
pcDNA3.1-lnc SFFD
pcDNA3.1-lnc SFFD +miR-202-3p mimic
siRNA NC
si-lnc SFFD
si-lnc SFFD +miR-202-3p inhibitor
Tubulin
Repetition 2
Repetition 3
pcDNA3.1
pcDNA3.1-lnc SFFD
pcDNA3.1-lnc SFFD +miR-202-3p mimic
siRNA NC
si-lnc SFFD
si-lnc SFFD +miR-202-3p inhibitor
pcDNA3.1
pcDNA3.1-lnc SFFD
pcDNA3.1-lnc SFFD +miR-202-3p mimic
siRNA NC
si-lnc SFFD
si-lnc SFFD +miR-202-3p inhibitor
HSD17B1
Repetition 2
Repetition 3
pcDNA3.1
pcDNA3.1-lnc SFFD
pcDNA3.1-lnc SFFD +miR-202-3p mimic
siRNA NC
si-lnc SFFD
si-lnc SFFD +miR-202-3p inhibitor
pcDNA3.1
pcDNA3.1-lnc SFFD
pcDNA3.1-lnc SFFD +miR-202-3p mimic
siRNA NC
si-lnc SFFD
si-lnc SFFD +miR-202-3p inhibitor
Tubulin

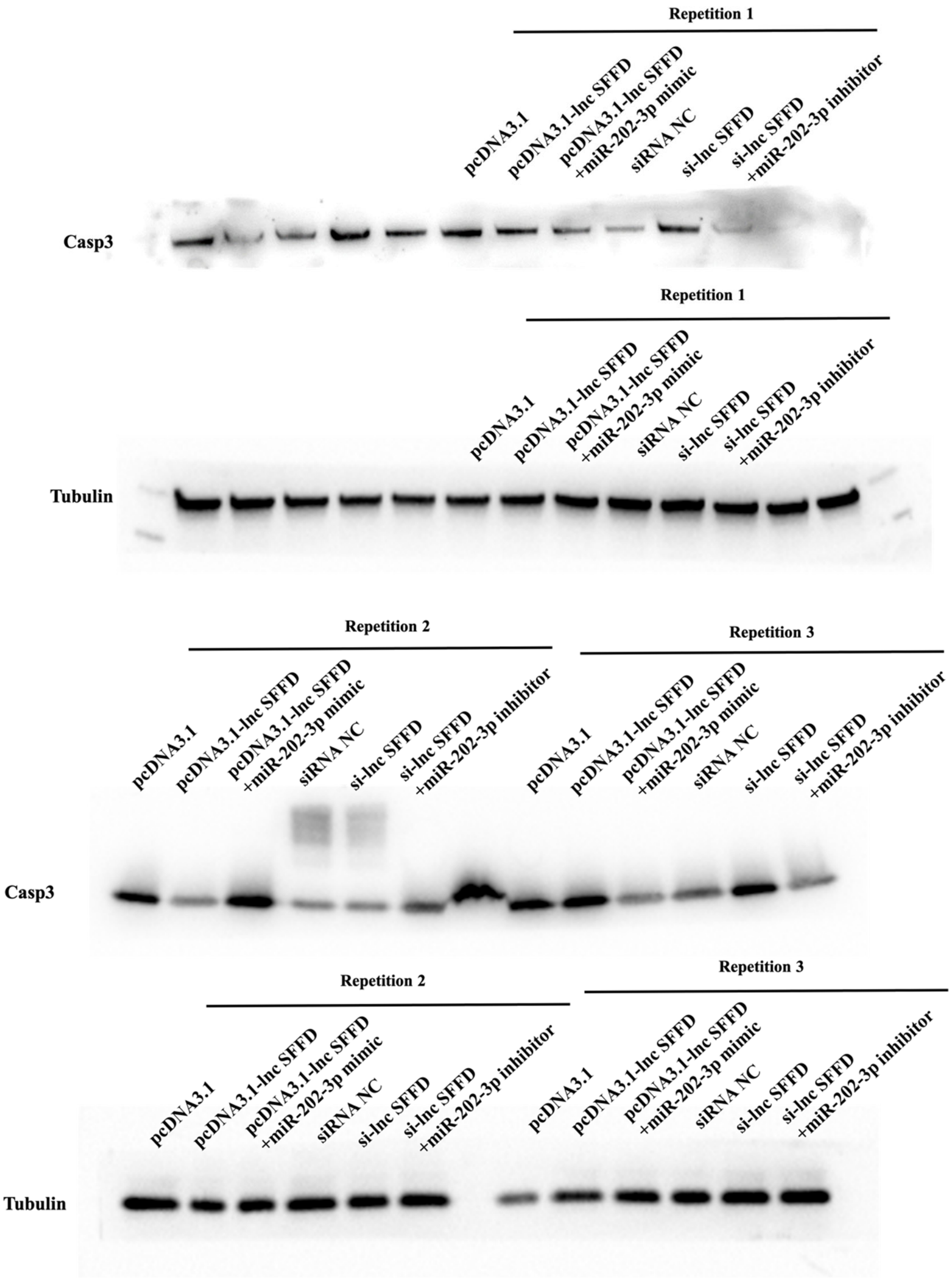
Repetition 1
pcDNA3.1
pcDNA3.1-lnc SFFD
pcDNA3.1-lnc SFFD +miR-202-3p mimic
siRNA NC
si-lnc SFFD
si-lnc SFFD +miR-202-3p inhibitor
Casp3
Repetition 1
pcDNA3.1
pcDNA3.1-lnc SFFD
pcDNA3.1-lnc SFFD +miR-202-3p mimic
siRNA NC
si-lnc SFFD
si-lnc SFFD +miR-202-3p inhibitor
Tubulin
Repetition 2
Repetition 3
pcDNA3.1
pcDNA3.1-lnc SFFD
pcDNA3.1-lnc SFFD +miR-202-3p mimic
siRNA NC
si-lnc SFFD
si-lnc SFFD +miR-202-3p inhibitor
pcDNA3.1
pcDNA3.1-lnc SFFD
pcDNA3.1-lnc SFFD +miR-202-3p mimic
siRNA NC
si-lnc SFFD
si-lnc SFFD +miR-202-3p inhibitor
Casp3
Repetition 2
Repetition 3
pcDNA3.1
pcDNA3.1-lnc SFFD
pcDNA3.1-lnc SFFD +miR-202-3p mimic
siRNA NC
si-lnc SFFD
si-lnc SFFD +miR-202-3p inhibitor
pcDNA3.1
pcDNA3.1-lnc SFFD
pcDNA3.1-lnc SFFD +miR-202-3p mimic
siRNA NC
si-lnc SFFD
si-lnc SFFD +miR-202-3p inhibitor
Tubulin

**5.The original blots shown in Figure 6F.**

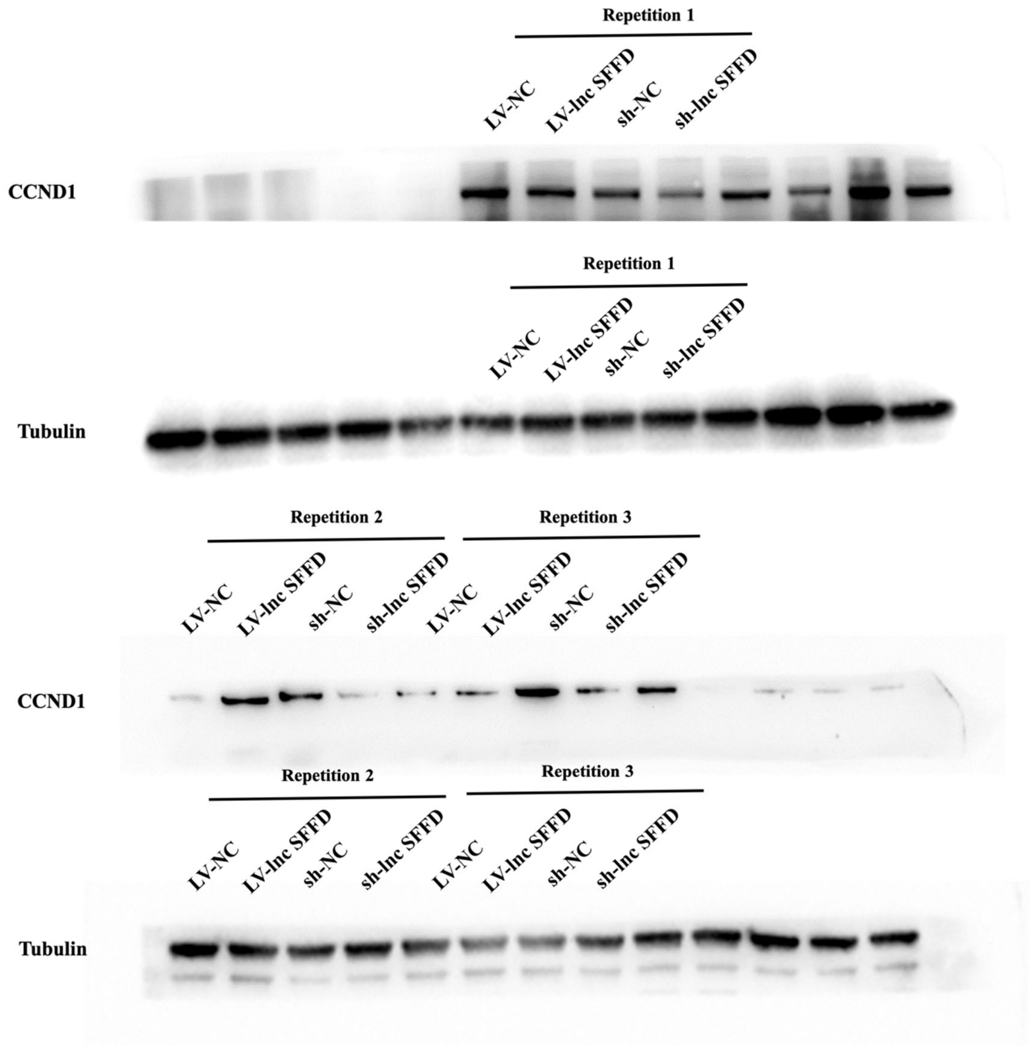

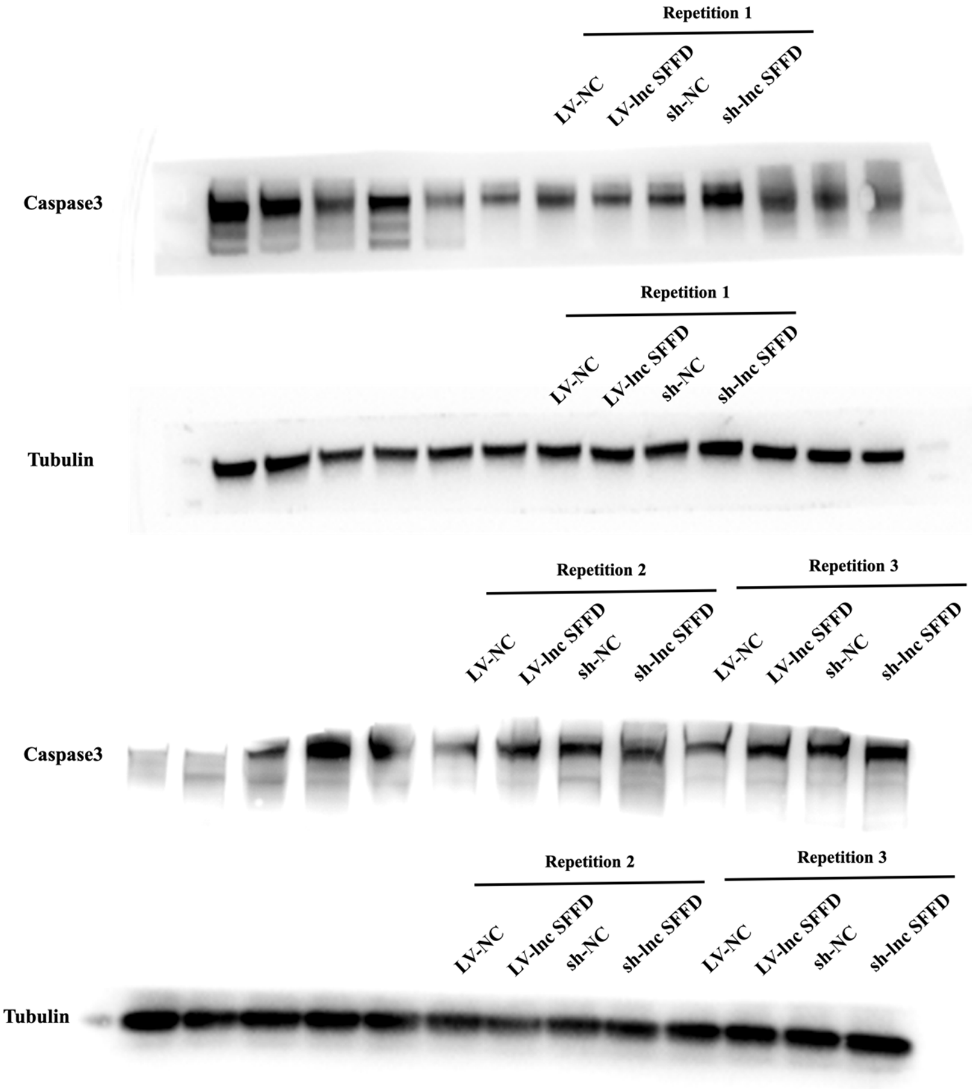
Repetition 1
LV-NC
LV-lnc SFFD
sh-NC
sh-lnc SFFD
Caspase3
Repetition 1
LV-NC
LV-lnc SFFD
sh-NC
sh-lnc SFFD
Tubulin
Repetition 2
Repetition 3
LV-NC
LV-lnc SFFD
sh-NC
sh-lnc SFFD
LV-NC
LV-lnc SFFD
sh-NC
sh-lnc SFFD
Caspase3
Repetition 2
Repetition 3
LV-NC
LV-lnc SFFD
sh-NC
sh-lnc SFFD
LV-NC
LV-lnc SFFD
sh-NC
sh-lnc SFFD
Tubulin

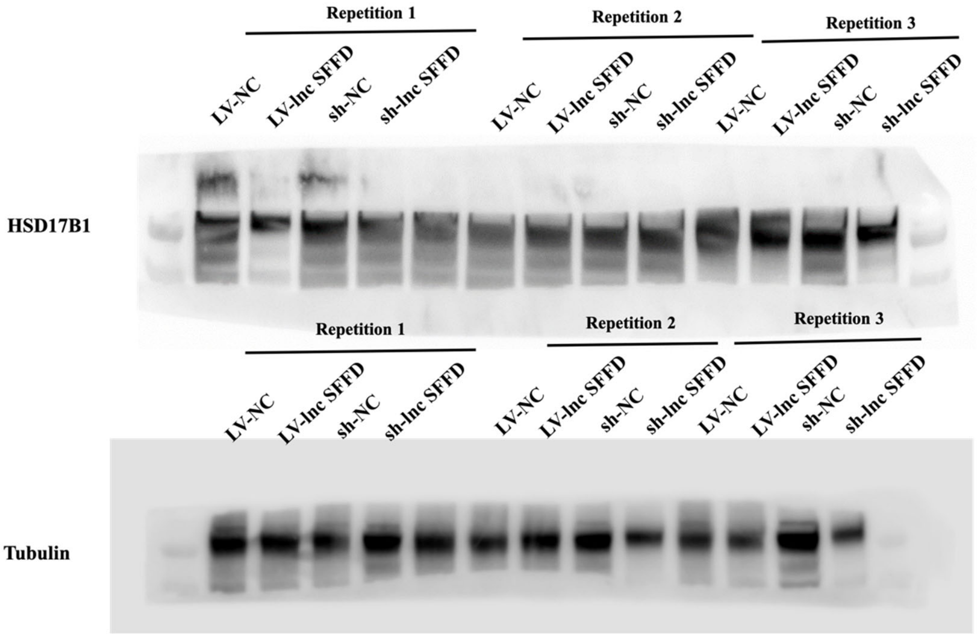
Repetition 1
Repetition 2
Repetition 3
LV-NC
LV-lnc SFFD
sh-NC
sh-lnc SFFD
LV-NC
LV-lnc SFFD
sh-NC
sh-lnc SFFD
LV-NC
LV-lnc SFFD
sh-NC
sh-lnc SFFD
HSD17B1
Repetition 1
Repetition 2
Repetition 3
LV-NC
LV-lnc SFFD
sh-NC
sh-lnc SFFD
LV-NC
LV-lnc SFFD
sh-NC
sh-lnc SFFD
LV-NC
LV-lnc SFFD
sh-NC
sh-lnc SFFD
Tubulin
